# Oral administration of a synthetic vinyl-ether plasmalogen normalizes open field activity in a mouse model of rhizomelic chondrodysplasia punctata

**DOI:** 10.1242/dmm.042499

**Published:** 2020-01-24

**Authors:** Wedad Fallatah, Tara Smith, Wei Cui, Dushmanthi Jayasinghe, Erminia Di Pietro, Shawn A. Ritchie, Nancy Braverman

**Affiliations:** 1 Department of Human Genetics and Pediatrics, Research Institute of the McGill University Health Center and McGill University, Montreal, QC H4A3J1, Canada; 2 Department of Medical Genetics, King Abdul-Aziz University, Jeddah, 21589 Saudi Arabia; 3 Med-Life Discoveries LP, Saskatoon, SK S7N2X8, Canada

**Keywords:** PPI-1040, Rhizomelic chondrodysplasia punctata, RCDP, Peroxisomal disorder, Plasmalogen

## Abstract

Rhizomelic chondrodysplasia punctata (RCDP) is a rare genetic disorder caused by mutations in peroxisomal genes essential for plasmalogen biosynthesis. Plasmalogens are a class of membrane glycerophospholipids containing a vinyl-ether-linked fatty alcohol at the sn-1 position that affect functions including vesicular transport, membrane protein function and free radical scavenging. A logical rationale for the treatment of RCDP is therefore the therapeutic augmentation of plasmalogens. The objective of this work was to provide a preliminary characterization of a novel vinyl-ether synthetic plasmalogen, PPI-1040, in support of its potential utility as an oral therapeutic option for RCDP. First, wild-type mice were treated with ^13^C_6_-labeled PPI-1040, which showed that the sn-1 vinyl-ether and the sn-3 phosphoethanolamine groups remained intact during digestion and absorption. Next, a 4-week treatment of adult plasmalogen-deficient *Pex7*^hypo/null^ mice with PPI-1040 showed normalization of plasmalogen levels in plasma, and variable increases in plasmalogen levels in erythrocytes and peripheral tissues (liver, small intestine, skeletal muscle and heart). Augmentation was not observed in brain, lung and kidney. Functionally, PPI-1040 treatment normalized the hyperactive behavior observed in the *Pex7*^hypo/null^ mice as determined by open field test, with a significant inverse correlation between activity and plasma plasmalogen levels. Parallel treatment with an equal amount of ether plasmalogen precursor, PPI-1011, did not effectively augment plasmalogen levels or reduce hyperactivity. Our findings show, for the first time, that a synthetic vinyl-ether plasmalogen is orally bioavailable and can improve plasmalogen levels in an RCDP mouse model. Further exploration of its clinical utility is warranted.

This article has an associated First Person interview with the joint first authors of the paper.

## Abstract (precis)

**Summary:** This article shows, for the first time, that a synthetic vinyl-ether plasmalogen is orally bioavailable and bioactive *in vivo* following administration in animals.

## INTRODUCTION

Rhizomelic chondrodysplasia punctata (RCDP) is a heterogeneous group of genetic disorders with a prevalence estimated at less than 1 per 100,000 (Stoll et al., 1989). RCDP results from mutations in peroxisomal genes involved in plasmalogen phospholipid production and is clinically characterized by skeletal dysplasia (rhizomelia and chondrodysplasia punctata), congenital cataracts, and profound growth, motor and cognitive delays. Neurological manifestations commonly include seizures and delayed myelination on brain magnetic resonance imaging (Bams-Mengerink et al., 2013, 2006). Dramatically reduced life expectancy is common to RCDP patients; however, survival varies widely with the severity of symptoms. For the classic, severe phenotype, only 50% will survive beyond 6 years of age and nearly all succumb to the disease prior to adolescence (White et al., 2003). The vast majority of deaths (80%) are reported as secondary to respiratory problems (White et al., 2003). Patients with milder phenotypes have variable rhizomelia and have better survival, growth and developmental outcomes.

Approximately 90% of patients have mutations in *PEX7* (RCDP1) (Braverman et al., 1997; Motley et al., 1997; Purdue et al., 1997), encoding the PEX7 receptor responsible for importing alkylglycerone phosphate synthase (AGPS) into the peroxisome. The remainder are caused by mutations in one of three genes encoding the peroxisomal enzymes that initiate the plasmalogen biosynthesis pathway: glycerophosphate-O-acyltransferase (*GNPAT*, RCDP2), *AGPS* (RCDP3) and fatty alcohol reductase 1 (*FAR1*, RCDP4) (Buchert et al., 2014; Wanders et al., 1994, 1992). Recently, two families were described with specific mutation in the long isoform of *PEX5*, encoding the PEX5 receptor. This mutation resulted in defective PEX7 binding to PEX5 and was classified as RCDP type 5 (Barøy et al., 2015). Regardless of the causative mutation, RCDP types have similar phenotypes and there is a direct correlation between phenotypic severity and residual plasmalogen levels (Bams-Mengerink et al., 2013; Braverman et al., 2002; Duker et al., 2017; Itzkovitz et al., 2012).

Plasmalogens are a class of glycerophospholipids, characterized by a vinyl-ether bond linkage between a 16:0, 18:0 or 18:1 fatty alcohol and the glycerol backbone at the sn-1 position, a fatty acid at the sn-2 position, and a polar head group at the sn-3 position of the glycerol backbone. There are two major classes of plasmalogens based on the head group: choline plasmalogens (PlsCho) and the more frequent ethanolamine plasmalogens (PlsEtn). Biosynthesis of plasmalogens begins in the peroxisome by the creation of the ether bond, which is reduced to a vinyl-ether within the endoplasmic reticulum by the enzyme plasmanylethanolamine desaturase TMEM189 (Gallego-García et al., 2019). Plasmalogens constitute ∼20% of the total phospholipid content of cell membranes. In many tissues, the majority of the glycerophosphoethanloamine fraction is PlsEtn (Braverman and Moser, 2012; Brites et al., 2004). Plasmalogens are essential for the architecture of lipid membranes and are critical for physiological processes such as vesicular fusion (Dorninger et al., 2019; Glaser and Gross, 1994; Lohner et al., 1991), membrane protein activity (da Silva et al., 2014; Dorninger et al., 2019; Wood et al., 2011b) and protection against oxidation (Kuczynski and Reo, 2006; Luoma et al., 2015). Reduced PlsEtn levels are one of the main biochemical features of peroxisome biogenesis disorders, including RCDP and Zellweger spectrum disorders, and were also reported in other diseases such as Alzheimer's disease (Goodenowe et al., 2007; Han et al., 2001; Kou et al., 2011; Wood et al., 2010), Parkinson's disease (Dragonas et al., 2009; Fabelo et al., 2011), schizophrenia (Kaddurah-Daouk et al., 2012), Down syndrome (Murphy et al., 2000) and Gaucher disease (Moraitou et al., 2014).

Natural sources of plasmalogen are not readily available, and thus dietary approaches to increase plasmalogen deficiency in RCDP are not feasible. Plasmalogen extracts have been generated from animal and marine tissues and tested in rodent models of Alzheimer's disease (Che et al., 2018; Hossain et al., 2018; Sejimo et al., 2018; Yamashita et al., 2017) and human patients with mild Alzheimer's disease (Fujino et al., 2017). The animal studies showed an improvement in the cognitive impairment and reduction in the neuroinflammation associated with Alzheimer's disease.

Several RCDP mouse models have been generated to investigate plasmalogen deficiency and test therapeutic interventions, including plasmalogen augmentation. Synthetic and concentrated naturally occurring ether precursors (alkylglycerols) including PPI-1011 and batyl alcohol were evaluated in RCDP mouse models (Braverman et al., 2010; Brites et al., 2011; Wood et al., 2011a). We sought to develop and evaluate a proprietary synthetic plasmalogen replacement strategy, which provided the intact vinyl-ether bond, eliminating the need for *in vivo* metabolism. To date, there are no reported studies evaluating synthetic vinyl-ether-containing molecules, likely due to uncertainty around the oral bioavailability of vinyl ethers, in conjunction with their inherently difficult synthesis processes and formulation requirements.

**Fig. 1.**
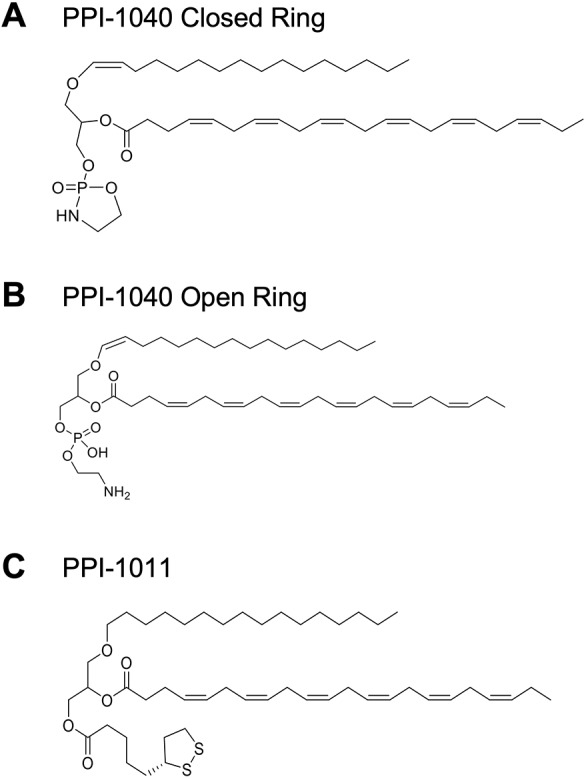
**Chemical structures of synthetic plasmalogens.** (A-C) The closed-ring version of PPI-1040 (A) is the form orally dosed to animals, which then undergo a spontaneous hydrolysis reaction upon exposure to an aqueous or acidic environment, resulting in the open-ring version (B), which is identical to endogenously occurring plasmalogen. PPI-1011 (C) is an ether analog to PPI-1040 that contains DHA at sn-2 and lipoic acid at sn-3 position of a palmitic ether glycerol.

In this paper, we demonstrate the feasibility of a synthetic PlsEtn compound (PPI-1040; Fig. 1A) containing a vinyl-ether-conjugated palmitic alcohol (C16:0) at the sn-1 position, docosahexaenoic acid (DHA, 22:6) at the sn-2 position, and a proprietary cyclic phosphoethanolamine group that increases the molecule's stability at the sn-3 position. Upon exposure to an aqueous environment, the cyclic phosphoethanolamine opens and the compound readily converts to, and becomes indistinguishable from, the matching endogenous plasmalogen species (Fig. 1B). Since PPI-1040 already contains the vinyl-ether bond, there is no requirement for any enzymatic metabolism *in vivo*. We hypothesize that this will be advantageous over ether-based precursors, and demonstrate herein the oral bioavailability, endogenous metabolism and functional effects of treatment with PPI-1040 in comparison to the ether precursor PPI-1011 (Fig. 1C) in the recently developed RCDP1 ‘*Pex7*^hypo/null^’ mouse model. The mice were generated to provide a model of intermediate severity between the *Pex7* hypomorphic model (Braverman et al., 2010) and the *Pex7* null (Brites et al., 2003).

## RESULTS

### Stability of PPI-1040 at acidic pH

**Fig. 2.**
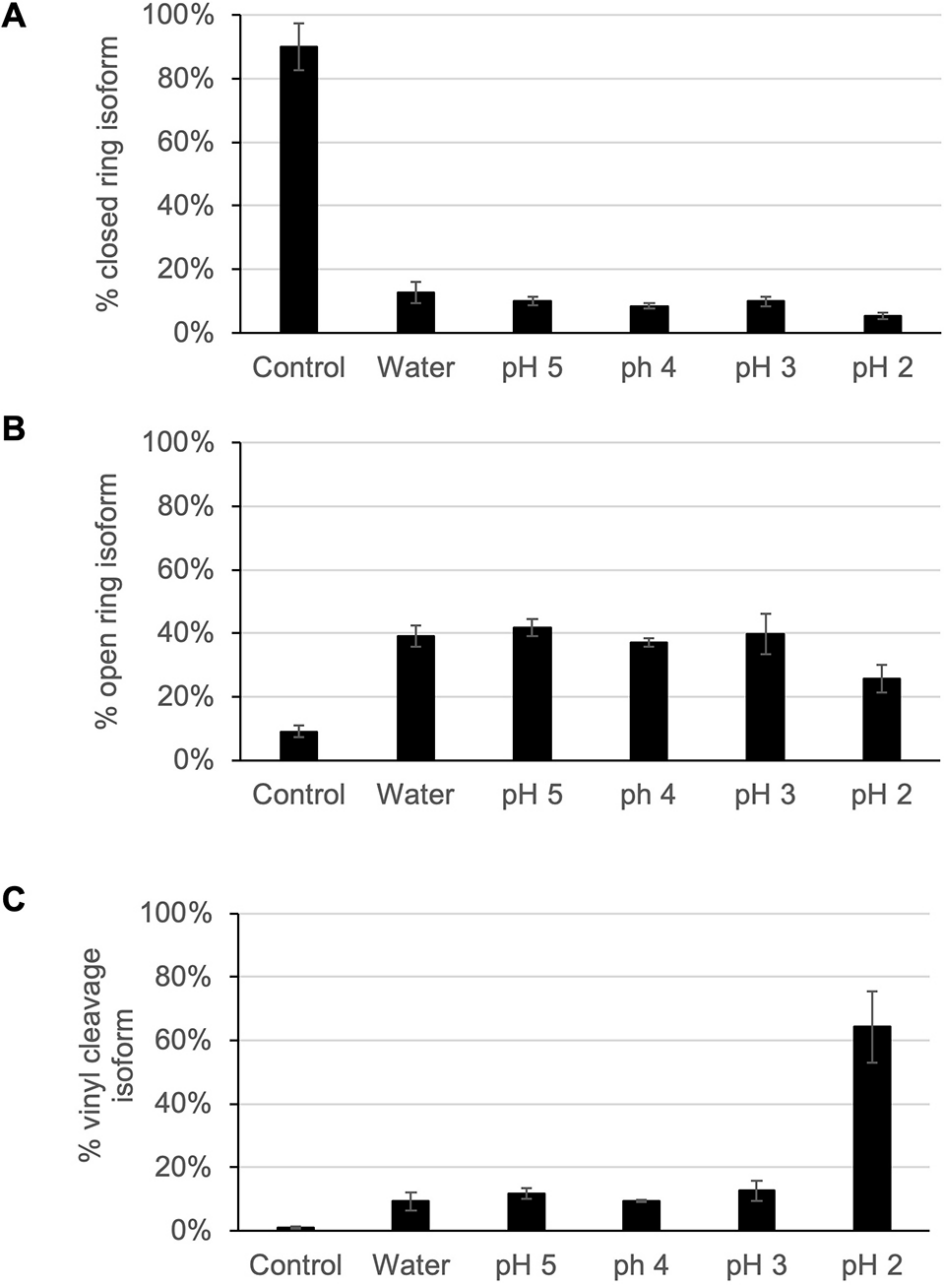
**Stability of PPI-1040 under increasingly acidic conditions as detected by HPLC FI-MS/MS.** (A) The closed-ring fully intact isoform of PPI-1040 was present at high levels in the formulated control sample, but was dramatically reduced upon exposure to water or acid. (B) Hydrolysis of the cyclic phosphoethanolamine group on PPI-1040 results in the open-ring isoform, which was the prominent form molecule in formulations exposed to water or acids of pH 3-5. (C) The vinyl-ether group at sn-1 is susceptible to cleavage under acidic conditions. Loss of the sn-1 group from open-ring PPI-1040 was monitored to detect vinyl cleavage, which was prominent at pH 2. Mean±s.d., *n*=3.

To confirm the viability of PPI-1040 as an oral therapy and verify its stability during the acidity associated with digestion, we exposed PPI-1040 to increasingly acidic conditions (pH 1-5) and monitored the levels of the intact compound, the open-ring isoform and the most likely degradation product (vinyl cleavage). Under the control conditions (formulated in Neobee M-5 with 0.1% thioglycerol), after 1 h, PPI-1040 remains in the intact closed-ring conformation (Figs 1A and 2A), with only a small amount of open ring detectable (Figs 1B and 2B). As designed, upon exposure to an aqueous environment, the cyclized phosphoethanolamine group at sn-3 readily opened, with levels of the closed-ring form being very low (<15%) with all treatments following a 1-h incubation (See Figs 1A and 2A). The open-ring version of PPI-1040, which is identical to endogenous PlsEtn 16:0/22:6, was the prominent species in the water treatment and the solutions of pH 3-5 (Figs 1B and 2B). The open-ring isoform of PPI-1040 lacking the sn-1 vinyl-ether alcohol (which would result from cleavage at the vinyl-ether bond), was minimal in the control, water and pH 3-5, suggesting that under these conditions there is little degradation at the sn-1 position (Fig. 2C). At a pH of 2, there was a reduction in the detection of the open-ring plasmalogen species and a corresponding increase in the amount of vinyl cleavage occurring, suggesting that at this pH the vinyl bond is compromised (Fig. 2B,C). At pH 1, all three species measured were at the limits of detection, suggesting that further degradation of the molecule was occurring.

### Oral bioavailability and metabolism of ^13^C-labeled PPI-1040 in wild-type C57BL/6 mice

**Fig. 3.**
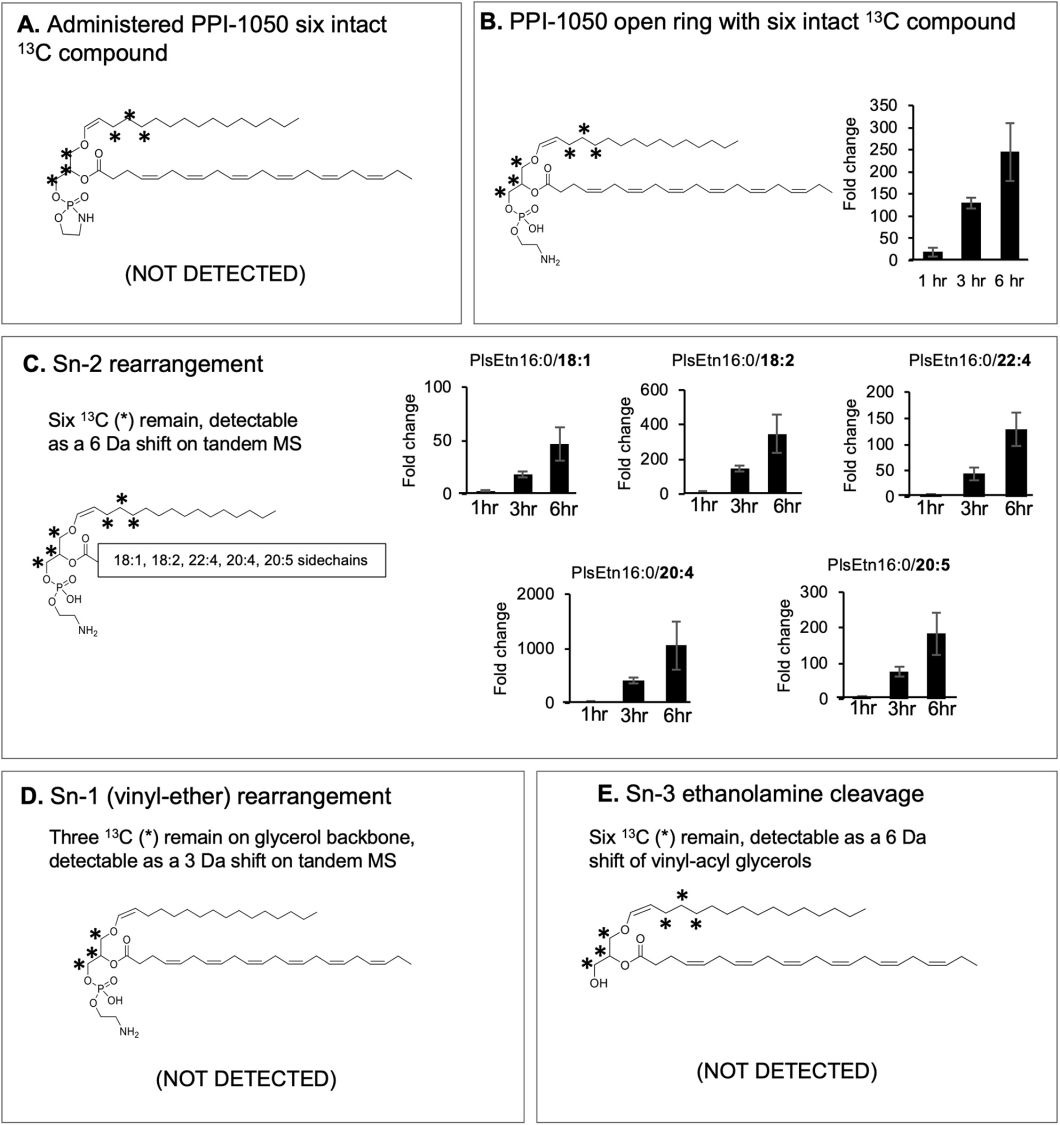
Outline of the possible metabolic conversions of ^13^C_6_-PPI-1050, including the number of ^13^C labels (*) and the mass shift expected for each product. (A) Following ingestion, the closed-ring version of PPI-1050 was not detectable in plasma. (B) PPI-1050 converts to the open-ring version following hydrolysis of the phosphoethanolamine ring, resulting in ^13^C_6_ -16:0/22:6 plasmalogen (PlsEtn). The graph denotes the fold change in the metabolite level relative to the vehicle group at each time point, illustrating a time-dependent increase. (C) Cleavage and remodeling of sn-2 would result in ^13^C_6_ -PlsEtn with differing sn-2 fatty acids, which were detected and mirrored the time-dependent increase seen in the target. (D) Cleavage and remodeling of the sn-1 group of PPI-1040 would result in ^13^C_3_-PlsEtn, which was not detected. (E) Cleavage of the sn-3 would result in ^13^C_6_ vinyl-acyl glycerols, which were not detected. Mean±s.d., *n*=3.

Oral bioavailability and uptake following absorption was investigated by dosing wild-type mice with PPI-1050, an isotopic version of PPI-1040. PPI-1050 was synthesized with six ^13^C labels, three strategically placed on each side of the vinyl-ether bond (Fig. 3A). We confirmed that there was no background signal in mouse serum at this mass shift by flow injection–tandem mass spectrometry (FI-MS/MS). This ^13^C configuration allowed us to interrogate various plasmalogen species and related molecules in plasma in order to conclude whether there was any *in vivo* (endogenous) rearrangement of PPI-1050 taking place up to 6 h following a single dose of 100 mg/kg. As expected, PPI-1050, containing the closed phosphoethanolamine ring intact, was not detected (Fig. 3A), while the open-ring version was readily detectable, with levels increasing in a time-dependent manner, confirming that the predominant form in plasma is the open ring (Fig. 3B). Removal of the sn-2 fatty acid group of plasmalogens by plasmalogen-selective phospholipase A2 (PLA2; also known as PlsEtn-PLA2) has been reported, and subsequently leads to the incorporation of other fatty acid groups at sn-2 (Balsinde et al., 2002; Farooqui and Horrocks, 2001; Horrocks and Sharma, 1982). We therefore assayed for plasmalogens, which would result from the vinyl bond remaining intact (maintaining the ^13^C_6_ mass shift), with the most commonly occurring sn-2 fatty acids. All sn-2 substitutions measured mirrored the time-dependent increase seen with the target PlsEtn (Fig. 3C). Overall abundance of the various PlsEtn species mirrored the endogenous distribution of the respective sn-2 fatty acid in normal plasma. We also assayed for two other combinations of resulting glycerolipid metabolic conversions: one where the sn-1 vinyl-ether bond would be cleaved (Fig. 3D) resulting in the loss of the three sn-1 ^13^C, and one where the sn-3 ethanolamine would be cleaved resulting in vinyl-acyl glycerols (Fig. 3E). There were no detectable signals for metabolites containing only three ^13^C labels (Fig. 3B), indicating no metabolism or rearrangement of the sn-1 vinyl-ether position, as well as no detectable metabolites containing six ^13^C labels but lacking the sn-3 ethanolamine. These results suggest stability of both the vinyl-ether and glycerophosphoethanolamine bonds during digestion and absorption.

### Oral bioavailability and pharmacokinetic comparison of vinyl-ether- versus ether-containing molecules in *Pex7*^hypo/null^

*Pex7*^hypo/null^ mice exhibit reduced plasmalogen levels, early cataracts, decreased growth and hyperactivity. These mice were treated for 4 weeks with ether plasmalogen precursor PPI-1011, synthetic vinyl-ether plasmalogen PPI-1040 or vehicle at 50 mg/kg per day (see Materials and Methods), and plasma and tissue PlsEtn levels were determined. A concurrent *Pex7*^WT/WT^ untreated cohort was included to compare to wild-type plasmalogen levels. Animal weights were recorded weekly. We observed no improvement in weights in the treatment groups compared to the vehicle-treated mice (Fig. S1). Cataracts were monitored by visual inspection and did not show improvement with treatment.

**Fig. 4.**
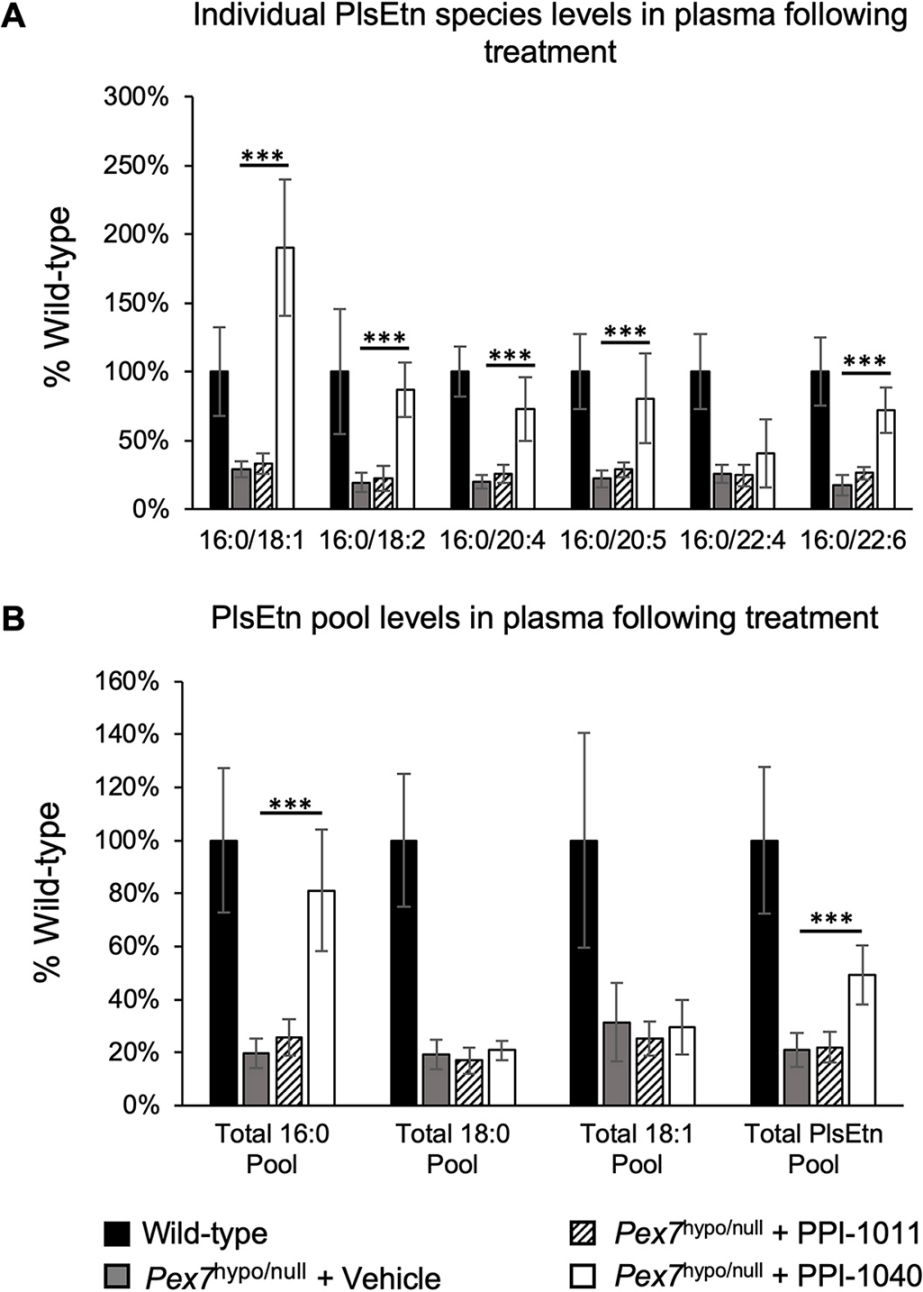
**Plasma PlsEtn levels in wild-type and *Pex7*^hypo/null^ mice treated with vehicle, PPI-1011 or PPI-1040 for 4 weeks at 50 mg/kg.** (A) Levels of the most abundant 16:0 (sn-1) plasmalogen species. (B) PlsEtn pools of the three major sn-1 fatty alcohols, as well as the total PlsEtn plasmalogen pool. All groups were significantly decreased in the vehicle relative to wild-type control. For the two treatment groups relative to the vehicle, PPI-1011 showed no significant augmentation of plasmalogens, whereas PPI-1040 significantly increased all 16:0 plasmalogen species measured except for 16:0/22:4. Levels are presented as the mean percentage of wild-type levels±s.d., *n*=4-6. Statistical analysis was performed using one-way ANOVA with Tukey's Honest Significant Differences post-hoc test, ***P<0.001.

Vehicle-treated *Pex7*^hypo/null^ mice showed significantly decreased plasma levels of all plasmalogens measured, averaging ∼25% of wild-type control levels (Fig. 4A). Treatment with PPI-1011 did not result in augmentation of any plasmalogen species in plasma, while treatment with PPI-1040 augmented levels of most PlsEtn species to near wild-type levels, with the exception of PlsEtn 16:0/22:4, which did not improve (Fig. 4A). Plasmalogens containing 18:0 and 18:1 at sn-1 were also measured, but, as anticipated, the alkyl group at the sn-1 position of the plasmalogen is not interconvertible (Das and Hajra, 1988; Dorninger et al., 2015), and no augmentation was observed in these species since the treatments were with a C16:0 plasmalogen (Fig. 4B). Because 16:0 (sn-1) represents over half the PlsEtn pool, treatment with PPI-1040 alone was able to restore total PlsEtn levels to ∼50% of control levels (Fig. 4B). No augmentation of any vinyl-acyl glycerol species, including 16:0/22:6, was observed (data not shown), consistent with our ^13^C-labeled results, indicating that there is no significant loss of the sn-3 phosphoethanolamine *in vivo*.

**Fig. 5.**
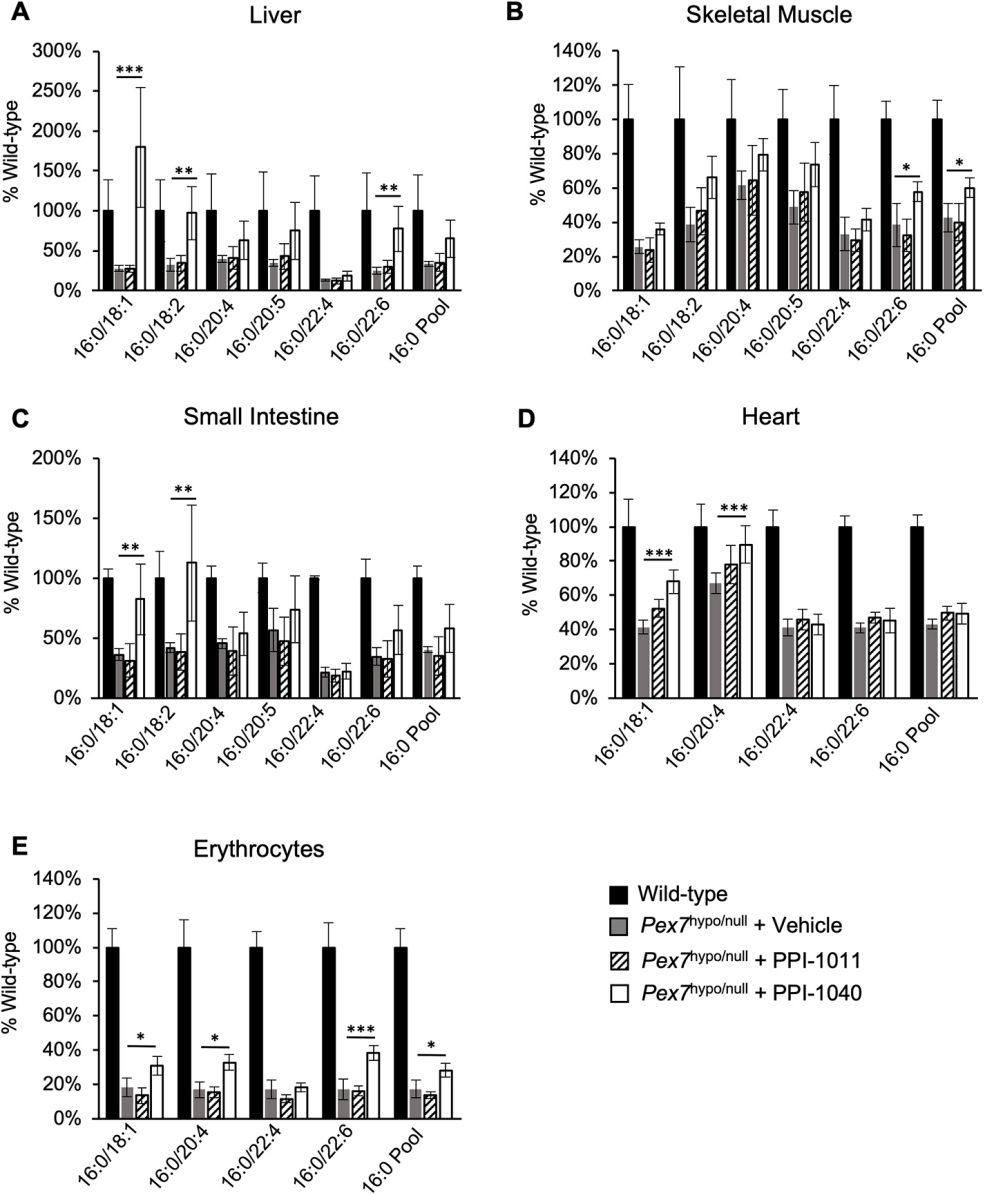
**Tissue plasmalogen levels in wild-type and *Pex7*^hypo/null^ mice treated with vehicle, PPI-1011 or PPI-1040.** (A-E) PPI-1011 treatment did not increase plasmalogen levels in any tissues analyzed. PPI-1040 treatment resulted in increased plasmalogen levels in the liver (A), skeletal muscle (B), small intestine (C), heart (D) and erythrocytes (E). Levels are presented as the mean percentage of wild-type levels±s.d. Statistical analysis was performed using one-way ANOVA with Tukey's Honest Significant Differences post-hoc test, *n*=4-6, *P<0.05, **P<0.01. ***P<0.001.

In addition to plasma, a variety of peripheral tissues, as well as brain, were analyzed for PlsEtn levels. The focus was to evaluate peripheral tissues, which could potentially benefit from postnatal plasmalogen augmentation. Consistent with plasma, PPI-1011 did not significantly increase the levels of any PlsEtn species in any of the tissues tested (Fig. 5; Fig. S2). PPI-1040 increased PlsEtn levels to varying degrees in some peripheral tissues, with augmentation observed in the liver, skeletal muscle, small intestine, heart and erythrocytes. In the liver and skeletal muscle, the levels of all 16:0 PlsEtn species (except for 16:0/22:4) and the 16:0 plasmalogen pool showed a trend towards improvement following PPI-1040 treatment (Fig. 5A,B). The 16:0/18:1, 16:0/18:2 and 16:0/22:6 species were significantly increased in liver (Fig. 5A), and the 16:0/22:6 species was significantly increased in the skeletal muscle (Fig. 5B). Small intestine showed a high degree of inter-animal variability; the levels of several species trended towards augmentation in the small intestine, but only 16:0/18:1 and 16:0/18:2 reached significance (Fig. 5C). The heart showed significant improvement in the 18:1 and 20:4 species (Fig. 5D). Similar to the plasma, erythrocytes showed augmentation in all species measured, except for 22:4 (Fig. 5E). PlsEtn containing 18:0 and 18:1 at sn-1 were not augmented in any tissues (data not shown). Lung, kidney, cortex and cerebellum did not display significant PlsEtn increases following either PPI-1011 or PPI-1040 treatment (Fig. S2).

### Effect of synthetic plasmalogen treatment on the behavior of Pex7^hypo/null^ mice

**Fig. 6.**
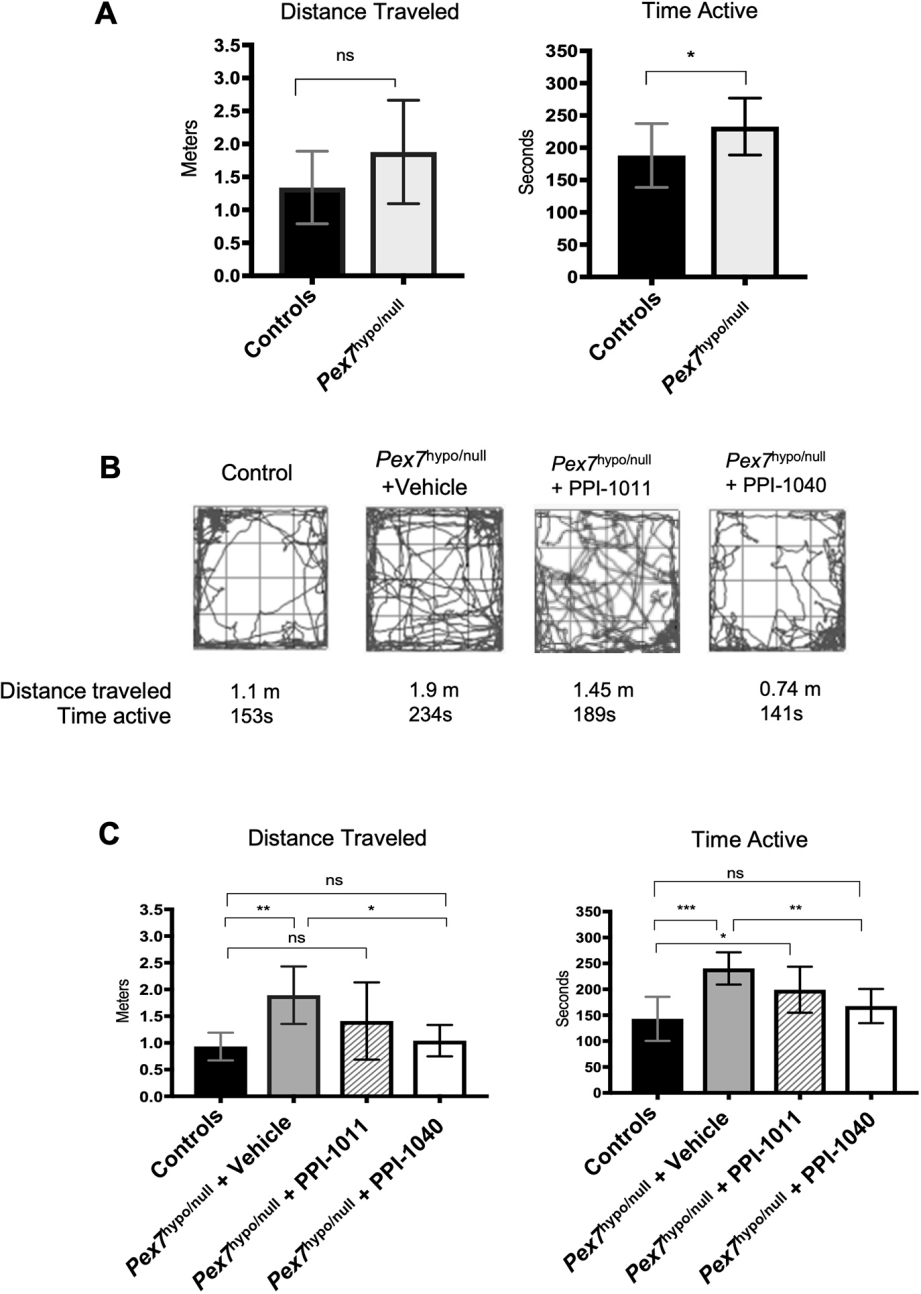
**Results of the open field tests from *Pex7* controls and *Pex7*^hypo/null^ at baseline and following treatment with vehicle, PPI-1011 or PPI-1040.** (A) Increased activity level of *Pex7*^hypo/null^ mice at baseline as measured by distance traveled (meters) and time active (seconds). (B) Representative tracking data of treated animals in the open field. (C) Open field test at the end of treatment showed significant hyperactivity in *Pex7*^hypo/null^ vehicle and PPI-1011 treated groups compared to controls. Normalization of the hyperactivity behavior to the level of the control mice was observed only in PPI-1040-treated *Pex7*^hypo/null^ mice. Statistical analysis was performed using one-way ANOVA with Tukey's Honest Significant Differences post-hoc test. *P<0.05, **P<0.01, ***P<0.001. ns, not significant.

**Fig. 7.**
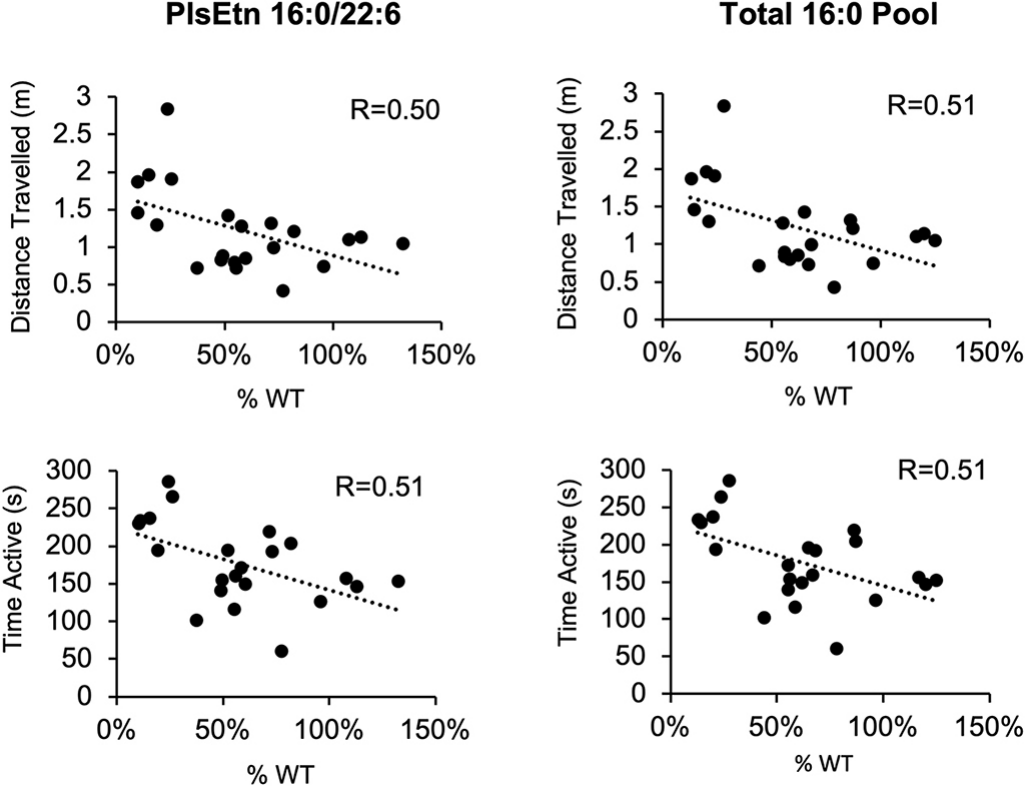
**Correlation between plasma PlsEtn levels and behavior.** Plasma PlsEtn 16:0/22:6 levels strongly correlated with distance traveled (R=0.50, *F*=6.67, *P*=0.015) and time active (R=0.51, *F*=7.08, *P*=0.015) (left column). Total 16:0 PlsEtn pool levels also correlated with distance traveled (R=0.51, *F*=6.92, *P*=0.016) and time active (R=0.51, *F*=7.10, *P*=0.014) (right column). *n*=10 controls, *n*=6 *Pex7*^hypo/null^ mice per group. Statistical analysis was performed by basic linear regression analysis. WT, wild type.

All four experimental mouse cohorts were subjected to the open field test before treatment (baseline) and at the end of the treatment period. A similar RCDP2 mouse model, based on a knockout of the *Gnpat* gene, had previously been demonstrated to be hyperactive in the open field test (Dorninger et al., 2019). In the untreated *Pex7*^hypo/null^ mice, we also demonstrated similar hyperactivity in the open field test in both young and older mice (data not shown). In this experiment at baseline, *Pex7*^hypo/null^ mice showed significantly increased activity compared to their matched controls (Fig. 6A). At the end of treatment, both vehicle and PPI-1011-treated *Pex7*^hypo/null^ mice displayed increases in time active (seconds) compared to controls (Fig. 6B,C). A significant difference in distance traveled was also observed in the vehicle-treated animals compared to controls (Fig. 6B,C). In contrast, PPI-1040 treatment normalized the hyperactive phenotype as assessed by both time active and distance traveled (Fig. 6B,C). In addition, plasma plasmalogen levels were shown to correlate with this behavioral phenotype. Comparing the control, vehicle and PPI-1040 animals, the plasma levels of the target 16:0/22:6 plasmalogen strongly correlated with both distance traveled (R=0.50, *F*=6.67, *P*=0.018) and time active (R=0.51, *F*=7.08, *P*=0.015) (Fig. 7). Total 16:0 plasmalogen levels in the plasma allowed for an assessment of the total pool of plasmalogens augmented following treatment, and were also found to correlate with both distance traveled (R=0.51, *F*=6.92, *P*=0.016) and time active (R=0.51, *F*=7.10, *P*=0.014) (Fig. 7).

## DISCUSSION

RCDP pathophysiology is due to a deficiency in the biosynthesis of plasmalogens, the rate-limiting step being the generation of the alkyglycerol species within the peroxisome. Thus, previous treatment approaches have focused on administration of plasmalogen precursors to bypass the peroxisomal steps of plasmalogen biosynthesis. Most notably *in vivo* studies have investigated the use of natural alkyl-ether glycerols (batyl alcohol) (Braverman et al., 2010; Brites et al., 2011) and synthetic ether glycerolipids (PPI-1011) (Wood et al., 2011a) in RCDP mouse models.

In this paper, we describe a novel synthetic plasmalogen, PPI-1040, which, unlike previously evaluated molecules, contains an intact vinyl-ether bond, eliminating the need for *in vivo* enzymatic metabolism. We showed that the PPI-1040 plasmalogen compound is orally bioavailable, and that both the sn-1 vinyl-ether and the sn-3 phosphoethanolamine bonds remain intact during digestion, absorption and circulation throughout the body. The efficacy of this novel synthetic plasmalogen to augment plasmalogen levels in *Pex7*-deficient mice was also evaluated.

### Stability and bioavailability of PPI-1040

Previous studies showed that the vinyl-ether bond in a plasmalogen molecule is unstable, highly acid labile and subject to cleavage through various free-radical-mediated processes (Stadelmann-Ingrand et al., 2001; Yavin and Gatt, 1972). In this study, we improved the vinyl-ether stability in PPI-1040 by introducing a proprietary cyclic phosphoethanolamine at sn-3 that reduces the polarity of the molecule, thereby protecting the vinyl bond from interacting with reactive molecules.

To ensure the feasibility of PPI-1040 as an oral therapeutic option, we examined the stability of the vinyl-ether bond in an acidic environment. The pH stability of PPI-1040 showed that the vinyl-ether was stable down to a pH of 3. Although the pH of the stomach in the fasting state can be as low as 2, it increases with food intake up to pH 5-7 (Dressman et al., 1990), suggesting that oral administration of a vinyl ether might be feasible. Increased absorption following oral administration of intact plasmalogens has previously been reported. In one study, Wistar rats were fed a purified plasmalogen source from bovine brain, and the vinyl-ether bond was detected in the total lipid fraction of plasma over 4 h (Nishimukai et al., 2003). Our study tested the *in vivo* oral bioavailability of a synthetic vinyl-ether molecule and further reinforced that an oral route of delivery was a viable option. We investigated the *in vivo* oral bioavailability and metabolism of PPI-1040 using the ^13^C-labeled isoform of PPI-1040, PPI-1050. The ^13^C labels were strategically placed within the backbone such that we could trace whether hydrolysis was occurring at the sn-1 vinyl-ether or the sn-3 phosphoethanolamine positions, or neither. We observed that the phosphoethanolamine ring opened as designed and the molecule absorbed intact. PLA2-mediated remodeling at the sn-2 position readily occurred as expected, and the sn-1 and sn-3 positions remained intact following oral administration. The short duration of the bioavailability study using labeled PPI-1040 (PPI-1050) limited pharmacokinetic characterization of PPI-1040 beyond 6 h.

### Augmentation of plasmalogen levels in *Pex7*^hypo/null^ mice

Oral administration of PPI-1040 in the *Pex7*^hypo/null^ mice at 50 mg/kg for 20 daily doses showed that PPI-1040 was absorbed as the open-ring form and resulted in augmentation in plasma of multiple target plasmalogen species containing vinyl-ether 16:0 at sn-1 to the point that the entire 16:0 plasmalogen pool was almost normalized to wild-type levels. This was consistent with the ^13^C PPI-1050 data showing incorporation into multiple 16:0 target species within hours of exposure. These findings unequivocally demonstrate that it is possible to orally dose with a vinyl-ether molecule and have the vinyl-ether bond remain intact as the glycerol-sn-1-sn-3 backbone is used to repopulate the endogenous pool, presumably by PLA2 (Balsinde et al., 2002; Farooqui and Horrocks, 2001; Horrocks and Sharma, 1982).

PPI-1040 also improved corresponding 16:0 PlsEtn levels to varying degrees in erythrocytes, liver, small intestine, skeletal and heart muscles of *Pex7*^hypo/null^ mice. Plasma and liver had the most robust elevation, while erythrocytes and other peripheral tissues such as skeletal muscle, small intestine and heart had more modest plasmalogen increases. No elevation was observed in the lung, kidneys or central nervous system (CNS). Although assessing tissue uptake was not the primary goal of this experiment, it was encouraging to observe augmentation in some peripheral tissues, such as small intestine and skeletal muscle. Since the majority of RCDP patients have gastrointestinal dysfunction (White et al., 2003), improving plasmalogen levels in the gastrointestinal tract could be clinically beneficial.

Vance (1990) suggested that plasmalogen synthesis in the liver is followed by transport of these newly synthesized plasmalogens within the circulation by lipoproteins. The author showed that lipoproteins secreted by cultured rat hepatocytes contained amounts of ethanolamine plasmalogens equivalent to that measured in rat serum (Vance, 1990). Thus, we hypothesize that if adequate plasmalogen augmentation in plasma can be achieved, it could be transported to tissues, although tissue uptake might be variable. Future studies examining chronic administration, and/or studies with radiolabeled precursors to determine whole-body absorption and distribution more sensitively, are warranted.

In order to directly compare equal doses of PPI-1040 to an ether precursor, we treated animals with PPI-1011, a similar compound but lacking the vinyl-ether bond at sn-1 and lipoic acid at sn-3. In this study, PPI-1011 failed to augment C16:0 plasmalogen levels in any of the tissues tested. While PPI-1011 was ineffective in the *Pex7*^hypo/null^ mice in this study, at a higher dose, labeled PP1-1011 was previously shown to incorporate into various tissues in the *Pex7* hypomorphic mouse model (Wood et al., 2011a). Batyl alcohol supplementation of *Pex7* null mice for several months at doses of 3000 mg/kg per day augmented C18:0 plasmalogen levels in all peripheral tissues, but not in the CNS, with only marginal increases in the peripheral nervous system (Brites et al., 2011).

### Functional improvement in response to plasmalogen supplementation

Behavioral assessments in RCDP mouse models are limited due to early development of cataracts, which impact the visual abilities of the mice. Hyperactivity in the open field test has been reproducible in different RCDP mouse models (Dorninger et al., 2019). Furthermore, it is a potentially clinically useful behavioral phenotype as it has been reported in patients with mild RCDP disease (Yu et al., 2013). To evaluate the effect of plasmalogen treatment on behavior, we examined the activity levels of *Pex7*^hypo/null^ mice in the open field test before and after therapeutic intervention. In the current study, the hyperactivity phenotype was observed in *Pex7*^hypo/null^ mice before treatment. Following the 20 days of exposure to PPI-1040, the activity of the *Pex7*^hypo/null^ mice was indistinguishable from that of controls. In contrast, hyperactive behavior did not improve in *Pex7*^hypo/null^ mice treated with PPI-1011 or vehicle. These results provide the first observation of a quantifiable improvement in a functional endpoint following augmentation with a vinyl-ether plasmalogen in deficient mice. Although the underlying mechanism of hyperactivity phenotype in *Pex7*-deficient mice is still under investigation, a recent study suggested a significant effect of impaired neurotransmission homeostasis on the hyperactive behavior seen in *Gnpat* null mice. The authors of this study concluded that the hyperactive behavior in *Gnpat* null mice could be a direct result of impaired synaptic vesicle function and reduced neurotransmitter levels (Dorninger et al., 2019).

Previous reports also showed functional effects of alkyl ether supplementation in the absence of augmentation. For example, an improvement in sciatic motor nerve conduction velocities was reported in *Pex7* null mice fed batyl alcohol (Brites et al., 2011). Several other studies showed functional nervous system improvement, but did not report associated plasmalogen levels. These include recovery of lipopolysaccharide (LPS)-mediated memory loss by the water maze test and reduced glial activation in the murine brain following 3 months of oral ingestion of plasmalogen extracted from chicken breast meat and provided as a suspension in drinking water to wild-type mice (Hossain et al., 2018). In parkinsonian monkeys, it was shown that 25 mg/kg of oral PPI-1011 supplementation improved the L-dopa-induced dyskinesia score compared to monkeys who did not receive PPI-1011 (Bourque et al., 2018; Grégoire et al., 2015). Furthermore, Malheiro et al. (2019) reported the ability of a short-chain (C14:0) alkyl glycerol, injected daily at 5 mg/kg starting at postnatal day 1 for 18 days, to improve myelination in the brain of the *Gnpat* null mouse model. Although plasmalogen levels were not directly measured in this study, these results strongly support incorporation of plasmalogens into CNS tissue. These results showing functional neurological improvement without simultaneous detectable increases in plasmalogen levels in nervous system tissues support the ability of plasmalogen supplementation to impact the CNS.

One possible explanation for what appears to be CNS-mediated effects in the absence of observable augmentation could be related to the potential technical challenges of sensitively measuring region-specific changes in the brain. Considering plasmalogen analysis of cortex, the results from homogenized samples largely reflect myelin-rich white matter, which is high in PlsEtn but has a slower turnover rate and different lipid profile from gray matter (Rosenberger et al., 2002). Therefore, small changes in white matter plasmalogen levels and/or changes in gray matter plasmalogens might be lost or masked among crude brain homogenates, especially if subtle changes in precise regions happen to be functionally relevant. It could also be that behavioral improvement is secondary to augmentation of plasmalogens in various peripheral tissues. In future experiments it would be desirable to dissect out regions of interest for PlsEtn analysis, as well as to directly measure molecular and cellular improvements in brain function. For example, neurotransmitter levels could be measured, as previous studies in *Gnpat* null mice indicated that plasmalogens are required for brain neurotransmitter homeostasis (Brodde et al., 2012).

Our study also provides the first analysis of plasmalogen subclasses and their response to treatment in *Pex7*-deficient mice by the use of liquid chromatography–tandem mass spectrometry (LC-MS/MS). This method precisely identifies different plasmalogen subspecies by identifying the specific groups at each position of the glycerol backbone: the fatty alcohol at sn-1, fatty acid at sn-2 and polar head group at sn-3. Prior studies used gas chromatography–mass spectrometry (GC-MS) to measure plasmalogen by detecting amounts of C16:0. C18:0 and C18:1 dimethyl acetal derivatives of the ether-linked alkenes, thus losing information on the specific subclasses at the sn-2 and sn-3 position (Steinberg et al., 2008).

In conclusion, we show that it is feasible to orally administer a synthetic vinyl-ether plasmalogen, and that the molecule stays intact during digestion, absorption and circulation within the body. Our data also support the hypothesis that a vinyl-ether backbone may be an option for the treatment of RCDP, given its ability to rescue plasmalogen levels and potentially impact functional endpoints. Further studies will be performed to more accurately and sensitively determine plasmalogen augmentation across all tissues, including CNS, as well as to investigate combined replacement of both 16:0 and 18:0 plasmalogens, and the potential benefits of earlier intervention.

## MATERIALS AND METHODS

### Drugs

PPI-1011, PPI-1040 and PPI-1050 (Figs 1 and 2A) were formulated in Neobee M-5 (Stepan Liquid Nutrition) with 0.1% thioglycerol (99%, Sigma-Aldrich) at a concentration of 10 mg/ml (PPI-1011 and PPI-1040) or 25 mg/kg (PPI-1050). PPI-1011 was stored at 4°C, while PPI-1040 and PPI-1050 were stored at −80°C due to reduced stability. Treatments with vehicle consisted of dosing animals with an equal volume of Neobee M-5 with 0.1% thioglycerol. Drug formulations were equilibrated to room temperature and oral gavage volumes were adjusted by mouse weight to ensure the indicated mg/kg dose. PPI-1011 and PPI-1040 were identified as intact molecules by LC-MS/MS.

### *In vitro* acid stability

To test the ability of the vinyl-ether bond in PPI-1040 to withstand the acidic nature of the stomach, the compound was exposed to different pH conditions. PPI-1040 was formulated into Neobee M-5 with 0.1% thioglycerol as outlined above. Formulated PPI-1040 was then either left untreated (control) or exposed to aqueous [high-performance liquid chromatography (HPLC)-grade water] or acidic (pH solutions) conditions. The pH solutions were made by serial 10-fold dilutions of 1 M HCl in HPLC-grade water. The final dilution was completed by adding a 20-μl aliquot of the acid solution to 200 μl of formulated PPI-1040, resulting in a pH range from 1 to 5 (*n*=3). Each mixture was vortexed at room temperature for 1 h, then re-suspended in ethyl acetate to obtain a 10 μl/ml solution, which was analyzed using LC-MS/MS using an Agilent Zorbax SB-CN column (5 μm, 4.6×150 mm) at 0.5 ml/min flow rate with the HPLC-grade solvents in mobile phase (A=water; B=methanol) at a gradient of A, 25% (0-7 min), 20% (7-10 min), 10% (10-12 min), 100% (12-15 min), 0% (15-20 min), 25% (20-25 min) in positive-multiple-reaction-monitoring (MRM) scan mode. The structures were distinguished using both tandem mass spectrometry (MS/MS) transitions and corresponding retention times (t_R_). We sought to assess the stability of two portions of the PPI-1040 structure, the phosphoethanolamine ring and the vinyl-ether bond at the sn-1 position. To assess the ability of the phosphoethanolamine ring to open we measured both the closed ring, intact PPI-1040, using m/z 730.5/607.5 appearing at 18.3 min, and the open phosphoethanolamine ring version of PPI-1040, using m/z 748.5/607.5 appearing at 17.7 min. To evaluate the stability of the vinyl-ether bond following opening of the ring, we measured a loss of the sn-1 fatty alcohol resulting from cleavage of the vinyl-ether bond using m/z 526.3/385.3 appearing at 6.6 min.

### Oral bioavailability study

PPI-1050, a ^13^C_6_-labeled version of PPI-1040, was used to evaluate the ability of the intact compound to cross the gut lining and enter the bloodstream. PPI-1050 contains ^13^C labels on the three glycerol carbons and three carbons of the sn-1 vinyl-ether-linked palmitic alcohol. Wild-type C57BL/6 mice were purchased from Charles River (St Constant, QC, Canada) and treated with a single oral dose of 100 mg/kg PPI-1050 or vehicle (Neobee M5+0.1% thioglycerol). Animals were sacrificed, and their blood was harvested by cardiac puncture at 1, 3 and 6 h post-treatment (*n*=3). Plasma was analyzed for the presence of labeled PlsEtn using FI-MS/MS in negative mode, while labeled vinyl-acyl glycerols were measured in the positive mode. The number of ^13^C labels in the predicted parent, together with the corresponding daughter ion, was used to determine the quantitative MS/MS transition pair. The measured transitions can be found in Table S1. This study was conducted under a Laval University (Quebec City, QC, Canada) animal-care-committee-approved protocol (#2017039-1).

### Treatment of *Pex7*^hypo/null^ animals

The *Pex7* hypomorphic allele was generated by inserting a neo cassette into intron 2 and lox P sites around exon 3. These mice were previously characterized and reported (B6;129S6-Pex7^tm2.1Brav^) (Braverman et al., 2010). The *Pex7* null allele was generated by crossing the hypomorphic mice with the general cre deleter strain B6.C-Tg(cmv-cre)1Cgn/J and then breeding to remove cre. The *Pex7*^hypo/null^ model (B6; 6NCrl; 129S6-Pex7^tm2.3Brav^) was generated by breeding the homozygous hypomorphic mice to mice heterozygous for the *Pex7* null allele.

*Pex7*^hypo/null^ mice (3-4 months) and control littermates, the compound heterozygote *Pex7*^WT/hypo^ (*n*=6) as well as matched *Pex7*^WT/WT^ (*n*=4) of both sexes were used in this study. We chose the age of 3-4 months because the original plasmalogen levels and behavioral testing were fully characterized in this age group. We used this intermediate model because its survival was better than that of the *Pex7* null and there was less variation in tissue plasmalogens compared to the original *Pex7* hypomorphic model. The open field test was performed at baseline, after which the mice were randomly assigned into three treatment groups: PPI-1040, PPI-1011 and vehicle controls, with *n*=6 mice per group. Drug was administered at 50 mg/kg per day by oral gavage 5 days per week (Monday to Friday) for 4 weeks. Animals were weighed weekly and observed for signs of distress. The open field test was repeated at the end of treatment. To avoid selection bias, data analysis was performed after study completion. Animals were sacrificed 24 h after the last dose. Tissues (liver, anterior thigh muscle, proximal small intestine, lung, kidney, cortex and cerebellum) and plasma were harvested and stored at −80°C until analysis. This study was conducted under a McGill University (Montreal, QC, Canada) animal-care-committee-approved protocol (#5538).

### The open field test

We used the open field behavioral test to evaluate general locomotor activity (Gould et al., 2009). The animal is placed inside a custom-made square, gray acrylic box measuring 40×40×40 cm and allowed to move freely for 5 min while being recorded by an overhead USB camera (model 60531, Stoelting Co., Wood Dale, IL, USA). Footage was analyzed by an automated tracking system (Any-maze Video Tracking Software, Stoelting Co., Wood Dale, IL, USA), for total distance traveled (meters) and activity time (mobility in seconds). Since we did not observe any behavioral differences between untreated *Pex7*^WT/WT^ and *Pex7*^WT/hypo^ in the open field test at baseline or endpoint, we grouped them together as untreated controls (*n*=10).

### Quantification of intact plasmalogens and plasmalogen metabolites by FI-MS/MS

Tissue samples from the liver, kidney, lung, skeletal muscle, small intestine, cortex and cerebellum were frozen by submersion in liquid nitrogen and then homogenized using a Covaris Cryoprep™, resulting in a fine powder. The homogenous powder was then aliquoted using anti-static polypropylene disposable milligram scoops (TWD Tradewinds) in 4-6 mg of tissue per 1.4-ml Thermo matrix tube. HPLC-grade water (50 μl) was added, and samples were snap frozen in liquid nitrogen and stored at −80°C until extraction. Tissue samples were equilibrated to room temperate and sonicated for 15 min in an ice bath before mixing at 2000 rpm for 5 min. Lipids were extracted into 600 μl ethyl acetate by vortexing at 1750 rpm for 1 h followed by a 10-min centrifugation at 1750 ***g*** to obtain a clear ethyl acetate layer. Tissue lipid extracts were diluted into an ethyl acetate stock (brain regions diluted 1:10, peripheral tissues 1:5) containing labeled internal standard [^13^C-PlsEtn (C_37_^13^C_6_H_74_NO_7_P)]. Water (40 μl) was added to the diluted extracts and samples were stirred at 1500 rpm for 1 h, followed by a 2-min centrifugation at 1750 ***g***.

Plasma extraction was performed on 20-μl aliquots in 1.4-ml Thermo matrix tubes. Lipids were extracted by adding HPLC-grade water (50μl) and ethyl-acetate-containing labeled internal standard [^13^C-PlsEtn (C_37_^13^C_6_H_74_NO_7_P)] at 0.2 µg/ml (500 μl) to each plasma sample, and then vortexed at 1750 rpm for 1 h followed by a 2-min centrifugation at 1750 ***g*** for phase separation.

A 100-μl aliquot of the ethyl acetate layer was analyzed by FI-MS/MS on an API4000™ mass spectrometer (Applied Biosystems) coupled with an Agilent 1100 HPLC pump and auto sampler. Each transition was scanned for 50 ms with a total acquisition time per sample of 2 min. Ethyl acetate:methanol:water ratio of 80:15:5 at a flow rate of 600 μl/min was used as the mobile phase. All standards and stable isotopes used were >95% pure and manufactured by Med-Life Discoveries LP, and solvents were HPLC grade. Samples were analyzed in triplicate to control for instrument variability. The measured transitions can be found in Table S2. Stable isotope ratios for each analyte were calculated by dividing the raw area of the analyte by the raw area of the ^13^C-PlsEtn standard; this value was then normalized to the wet weight (mg) of the tissue analyzed. These normalized values were used to calculate the level of each analyte relative to the wild-type controls to generate a percentage wild-type value.

### Quantification of intact plasmalogens by LC-MS/MS

Whole-blood samples were collected in EDTA blood collection micro-tubes. A separation of plasma from erythrocytes was made by centrifugation, at 23,000 ***g*** for 15 min at 4°C. The supernatant was transferred into freezing vials and stored at −80°C. The pellet containing red blood cells was washed three times with 1× phosphate buffered saline (PBS) and centrifuged for 10 min at 4°C, then stored at −80°C until extraction.

Heart tissue was homogenized in PBS using a mini pestle, and 20 volumes of 2:1 chloroform/methanol containing 0.05% butylhydroxytoluene were added to 50 µg of protein extract in a glass tube and incubated for 2 h on an orbital shaker at room temperature. Samples were centrifuged at 1100 ***g*** for 10 min, and the supernatant was transferred to a clean glass tube, washed with 0.2 volumes of purified water, then centrifuged for 5 min at 700 ***g*** at room temperature to separate the two phases. The upper phase was removed and the lower phase was washed with Folch theoretical upper phase (3:48:47 chloroform:methanol:water). Samples were mixed and centrifuged at 700 ***g*** for 5 min, and the upper phase was removed. The lower phase was dried under nitrogen and then in a vacuum dessicator for 30 min. The dried lipid was dissolved in 3:2 hexane:isopropanol containing 10 ng each of the internal standards, 16:0-D4 lyso-PAF (20.6 pmol) (Cayman Chemicals, Ann Arbor, MI, USA) and D4-26:0-lyso-PC (15.6 pmol) (Avanti Polar Lipids, Alabaster, AL, USA). Erythrocytes (10 μl) were incubated with 150 μl methanol containing 10 ng of each internal standard for 2 h at room temperature with shaking. Heart tissue and erythrocyte samples were filtered by centrifugation (Costar spin-X centrifuge tube filters, Corning, NY, USA) for 5 min. Filtrates were analyzed in Verex auto-sampler vials (Phenomenex, Torrance, CA, USA). A 2.1×50 mm, 1.7 µm chromatography column and a Waters (Milford, MA, USA) triple-quadrupole mass spectrometer (TQD) interfaced with an Acquity ultra-performance liquid chromatography (UPLC) system was used in positive-ion electrospray (ESI)-MS/MS ionization. HPLC-grade solvent systems were as follows: mobile phase A=54.5% water/45% acetonitrile/0.5% formic acid; mobile phase B=99.5% acetonitrile/0.5% formic acid, with both solutions containing 2 mM ammonium formate. Injections were made with initial solvent conditions of 85% mobile phase A/15% mobile phase B. The gradient went from 15% to 100% mobile phase B over a period of 2.5 min, and was held at 100% mobile phase B for 1.5 min before reconditioning the column back to 85% mobile phase A/15% mobile phase B for 1 min at a solvent rate of 0.7 ml/min. A column temperature of 35°C and an injection volume of 5 μl PlsEtn were detected by MRM transitions representing fragmentation of [M+H]+ species to m/z 339, 361, 389 and 385 for PE plasmalogen compounds with 18:1, 20:4, 22:4 and 22:6 at the sn-2 position, respectively.

### Statistical analysis

Data are presented as mean±s.d. Behavioral data and plasmalogen levels were compared using one-way ANOVA with a Tukey's Honest Significant Difference post-hoc test. Basic linear regression was used to compare plasmalogen levels and behavioral scores. A *P*-value less than 0.05 was considered statistically significant.

## Supplementary Material

Supplementary information
