# Supplementary material for: Oral administration of a synthetic vinyl-ether plasmalogen normalizes open field activity in a mouse model of rhizomelic chondrodysplasia punctata
Source: Dis Model Mech. 2020 Jan 24;13(1):dmm042499. doi: 10.1242/dmm.042499 (PMC6994958; doi:10.1242/dmm.042499)
Supplement: Supplementary information [file dmm-13-042499-s1.pdf]

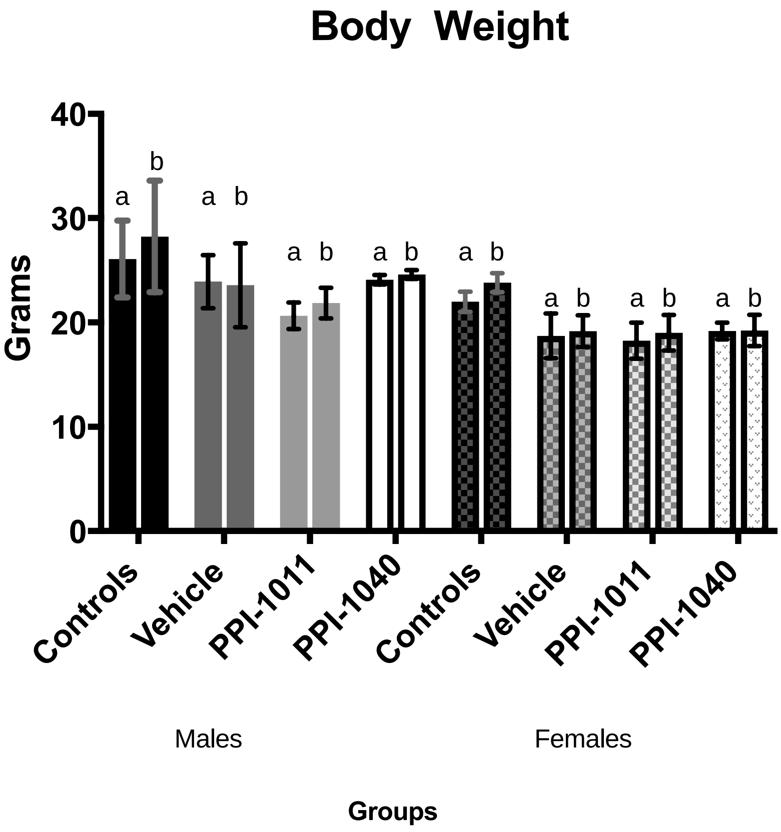

Figure S1. Body weight of Pex7$^{hypo/null}$ and control mice before (a) and after (b) the treatment. No significant increase in body weight of Pex7$^{hypo/null}$ mice after plasmalogen supplementation. Data were presented as mean ± SD.

Disease Models & Mechanisms • Supplementary information

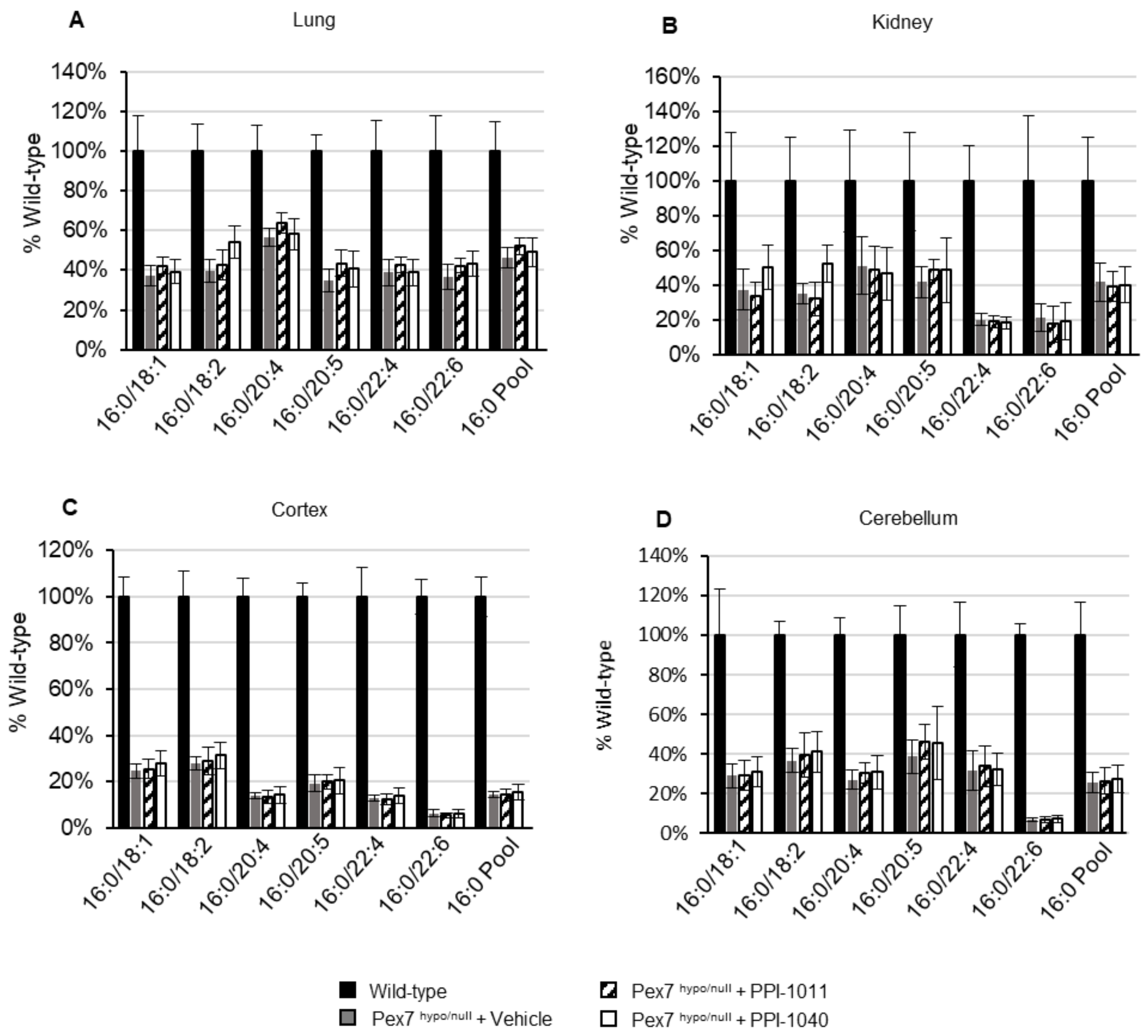

Figure S2. Tissue plasmalogen levels in wild-type and Pex7$^{hypo/null}$ mice treated with vehicle, PPI-1011 or PPI-1040. Neither PPI-1011 or PPI-1040 treatment increased plasmalogen levels in the lung (A), kidney (B), cortex (C), or cerebellum (D). Levels are presented as the mean percent of wild-type levels ± SD.

Table S1 – List of ^{13}C -labeled glycerolipids measured by tandem mass spectrometry.

Lipid Type	sn-1	sn-2	Glycerol	Analysis mode	MRM Transition
1050 closed ring	$^{13}C_3$ – 16:0	22:6	$^{13}C_3$	Positive	736.5/613.5
1050 open ring	$^{13}C_3$ – 16:0	22:6	$^{13}C_3$	Negative	752.5/327.2
PlsEtn	$^{13}C_3$ – 16:0	18:1	$^{13}C_3$	Negative	706.6/281.3
PlsEtn	$^{13}C_3$ – 16:0	18:2	$^{13}C_3$	Negative	704.5/279.2
PlsEtn	$^{13}C_3$ – 16:0	20:4	$^{13}C_3$	Negative	728.5/303.2
PlsEtn	$^{13}C_3$ – 16:0	20:5	$^{13}C_3$	Negative	726.5/301.2
PlsEtn	$^{13}C_3$ – 16:0	22:4	$^{13}C_3$	Negative	756.6/331.3
PlsEtn	16:0	18:1	$^{13}C_3$	Negative	703.5/281.3
PlsEtn	16:0	18:2	$^{13}C_3$	Negative	701.5/279.2
PlsEtn	16:0	20:4	$^{13}C_3$	Negative	725.5/303.2
PlsEtn	16:0	20:5	$^{13}C_3$	Negative	723.5/301.2
PlsEtn	16:0	22:4	$^{13}C_3$	Negative	753.6/331.3
PlsEtn	16:0	22:6	$^{13}C_3$	Negative	749.5/327.2
PlsEtn	18:0	18:1	$^{13}C_3$	Negative	731.6/281.3
PlsEtn	18:0	18:2	$^{13}C_3$	Negative	729.5/279.2
PlsEtn	18:0	20:4	$^{13}C_3$	Negative	753.6/303.2
PlsEtn	18:0	20:5	$^{13}C_3$	Negative	751.5/301.2
PlsEtn	18:0	22:4	$^{13}C_3$	Negative	781.6/331.3
PlsEtn	18:0	22:6	$^{13}C_3$	Negative	777.6/327.2
VAG	$^{13}C_3$ – 16:0	18:1	$^{13}C_3$	Positive	585.6/242.3
VAG	$^{13}C_3$ – 16:0	18:2	$^{13}C_3$	Positive	583.5/242.3

Disease Models & Mechanisms • Supplementary information

VAG	$^{13}C_3$ – 16:0	20:4	$^{13}C_3$	Positive	607.5/242.3
VAG	$^{13}C_3$ – 16:0	20:5	$^{13}C_3$	Positive	605.5/242.3
VAG	$^{13}C_3$ – 16:0	22:4	$^{13}C_3$	Positive	635.7/242.3
VAG	$^{13}C_3$ – 16:0	22:6	$^{13}C_3$	Positive	631.5/242.3
VAG	16:0	18:1	$^{13}C_3$	Positive	582.5/239.2
VAG	16:0	18:2	$^{13}C_3$	Positive	580.5/239.2
VAG	16:0	20:4	$^{13}C_3$	Positive	604.5/239.2
VAG	16:0	20:5	$^{13}C_3$	Positive	602.5/239.2
VAG	16:0	22:4	$^{13}C_3$	Positive	632.6/239.2
VAG	16:0	22:6	$^{13}C_3$	Positive	628.5/239.2

Disease Models & Mechanisms • Supplementary information

Table S2. List of ethanolamine plasmalogen analytes measured by tandem mass spectrometry.

Analyte	Molecular Formula	MRM transition
$^{13}C_6$-PlsEtn 16:0/22:6 (IS)	$C_{37}{}^{13}C_6H_{74}NO_7P$	752.5/327.2
PlsEtn 16:0/18:1	$C_{39}H_{76}NO_7P$	700.5/281.2
PlsEtn 16:0/18:2	$C_{39}H_{74}NO_7P$	698.5/279.2
PlsEtn 16:0/20:4	$C_{41}H_{74}NO_7P$	722.5/303.2
PlsEtn 16:0/20:5	$C_{41}H_{72}NO_7P$	720.5/301.2
PlsEtn 16:0/22:4	$C_{43}H_{78}NO_7P$	750.5/331.2
PlsEtn 16:0/22:6	$C_{43}H_{74}NO_7P$	746.5/327.2
PlsEtn 18:0/18:1	$C_{41}H_{80}NO_7P$	728.5/281.2
PlsEtn 18:0/18:2	$C_{41}H_{78}NO_7P$	726.5/279.2
PlsEtn 18:0/20:4	$C_{43}H_{78}NO_7P$	750.6/303.2
PlsEtn 18:0/20:5	$C_{43}H_{76}NO_7P$	748.5/301.3
PlsEtn 18:0/22:4	$C_{45}H_{82}NO_7P$	778.5/331.2
PlsEtn 18:0/22:6	$C_{45}H_{78}NO_7P$	774.5/327.2
PlsEtn 18:1/18:1	$C_{41}H_{78}NO_7P$	726.5/281.2
PlsEtn 18:1/18:2	$C_{41}H_{76}NO_7P$	724.5/279.2
PlsEtn 18:1/20:4	$C_{43}H_{76}NO_7P$	748.5/303.2
PlsEtn 18:1/22:4	$C_{45}H_{80}NO_7P$	776.5/331.2
PlsEtn 18:1/22:6	$C_{45}H_{76}NO_7P$	772.5/327.2

Disease Models & Mechanisms • Supplementary information
